# Surgical Treatment of Exophthalmic Goitre

**Published:** 1903-09-26

**Authors:** 


### Hospital Clinics and Medical Progress.

### SURGICAL TREATMENT OF EXOPHTHALMIC GOITRE.

The failure of medical treatment, in a large number of cases, to effect a cure or to relieve the patient from the distressing symptoms of Grave's disease has induced surgeons to make trial of many different operative procedures in these cases, among which may be mentioned partial thyroidectomy, ligature of the thyroid vessels, operations upon the cervical sympathetic nerves and exothyropexy. Of these methods the last needs no further notice beyond remarking that it consisted of laying bare the gland by free incisions, and leaving it exposed in the open wound covered only by dressings. The idea of this procedure being to produce atrophy of the gland. The operation is dangerous, disfiguring and unproductive of success. Ligature of the thyroid vessels has also proved disappointing, being almost as difficult as thyroidectomy and not so satisfactory as regards results. There remain therefore thyroidectomy and operations on the cervical sympathetic to be considered. Dr. Curtis[1] puts forward his own experiences and opinions on the respective merits of these two procedures. At the outset he calls attention to the well-known fact that surgical treatment in exophthalmic goitre, although capable of curing many cases which have not yielded to medicine, has very serious risks of its own, especially the danger of acute exacerbation of the symptoms known as "thyroidism," or thyroid poisoning, which may follow any operation in these patients whether the active interference concerns the thyroid gland itself or other parts of the body. Rehn's statistics are quoted in which 177 thyroidectomies by 37 operators show a mortality of 13·5 per cent. with 57·6 per cent. of cures and 26·5 per cent. of cases improved. Dr. Curtis has performed thyroidectomy 11 times with three deaths, every one of which was due to acute thyroidism, six cures, one improved, and one lost sight of. The operations were all performed by means of a transverse incision. The isthmus was sometimes crushed and ligated *en masse* with catgut, and sometimes divided with the cautery. Each of these methods was tried with the idea that it might prevent the occurrence of thyroid poisoning, but both failed to avoid this complication. Great gentleness was employed in handling the gland, to avoid the supposed danger of causing absorption of thyroid material. General anæsthesia with ether was employed. Owing to the frequency with which thyroidism had followed in his cases of thyroidectomy, Dr. Curtis ceased to perform that operation and began to perform excision of the cervical sympathetic instead, a procedure which he has now carried out in seven cases. But the results have not been much better than those of thyroidectomy, for he has had two deaths from acute thyroid poisoning and one death probably due to the anæsthetic. All the operations were done with ether anæsthesia, and both sides of the neck were dealt with in one operation except in one case. The incision was made along the anterior border of the sterno-mastoid in all but the first instance, and all three cervical ganglia were removed from each side of the neck. The results, so far as can be judged in the short time that has elapsed since the treatment was carried out, are as follows: three cured, one improved, and three died. It is obvious that the danger of acute thyroid poisoning, or thyroidism, following these operations in patients who have exophthalmic goitre is a very considerable one. In dealing with the symptoms he points out that the patient may have been put to bed in good condition after operation, having required no stimulant and apparently ready for a good convalescence. The temperature, however, will soon rise to 102° or 103° F. Usually in the cases ending fatally it will reach 103° or 104° F. in 24 hours and continue to rise to 106° or 107° F. With the rising temperature, the pulse will run up to 130, 150, or even 200 beats per minute. The face will be flushed and the respirations hurried and shallow. The patient will be nervous, tremulous and talking a good deal, though as a rule no anxiety will be displayed. Death may come quite suddenly. Morphia will quiet the restlessness, but no drug seems to be able to control the heart. One patient only of Dr. Curtis's cases survived the symptoms described over 24 hours. The temperature after operation in nearly every case was high, even rising to 103° F., although primary union of the wounds occurred. No leucocytosis was found with this rise of temperature. A careful study of each case has failed to throw much light upon the causes of the catastrophe, or to disclose any signs which might serve as warnings of an unhappy issue, unless the presence of albuminuria be proved to be a guide. In all five of his fatal cases Dr. Curtis found albumen in the urine after operation, but in only two of these was albuminuria discovered previous to operation. Of the cases which recovered four had albuminuria after operation. All these four patients had a marked rise of temperature and other unpleasant symptoms during convalescence. In the present state of our knowledge, therefore, it would appear that a slight trace of albumen in the urine of a patient suffering from exophthalmic goitre should be taken as a contra-indication to any operative interference.

[1] Annals of Surgery, August, 1903.

